# Galectin-3 Regulates Indoleamine-2,3-dioxygenase-Dependent Cross-Talk between Colon-Infiltrating Dendritic Cells and T Regulatory Cells and May Represent a Valuable Biomarker for Monitoring the Progression of Ulcerative Colitis

**DOI:** 10.3390/cells8070709

**Published:** 2019-07-12

**Authors:** Vladislav Volarevic, Natasa Zdravkovic, Carl Randall Harrell, Nebojsa Arsenijevic, Crissy Fellabaum, Valentin Djonov, Miodrag L. Lukic, Bojana Simovic Markovic

**Affiliations:** 1 Department for Microbiology and Immunology, Center for Molecular Medicine and Stem Cell Research, Faculty of Medical Sciences, University of Kragujevac, 69 Svetozar Markovic Street, 34000 Kragujevac, Serbia; 2 Department of Internal medicine, Faculty of Medical Sciences, University of Kragujevac, 69 Svetozar Markovic Street, 34000 Kragujevac, Serbia; 3 Center for Gastroenterology, Clinical Center Kragujevac, 30 Zmaj Jovina Stret, 34000 Kragujevac, Serbia; 4 Regenerative Processing Plant, LLC, 34176 US Highway 19 N Palm Harbor, Palm Harbor, FL 34684, USA; 5 Institute of Anatomy, University of Bern, 2 Baltzerstrasse, 3012 Bern, Switzerland

**Keywords:** ulcerative colitis, Galectin-3, dendritic cells, T regulatory cells, Kynurenine, Toll-like receptor-4, immunomodulation, biomarker

## Abstract

Galectin-3 regulates numerous biological processes in the gut. We investigated molecular mechanisms responsible for the Galectin-3-dependent regulation of colon inflammation and evaluated whether Galectin-3 may be used as biomarker for monitoring the progression of ulcerative colitis (UC). The differences in disease progression between dextran sodium sulphate-treated wild type and Galectin-3-deficient mice were investigated and confirmed in clinical settings, in 65 patients suffering from mild, moderate, and severe colitis. During the induction phase of colitis, Galectin-3 promoted interleukin-1β-induced polarization of colonic macrophages towards inflammatory phenotype. In the recovery phase of colitis, Galectin-3 was required for the immunosuppressive function of regulatory dendritic cells (DCs). Regulatory DCs in Galectin-3:Toll-like receptor-4:Kynurenine-dependent manner promoted the expansion of colon-infiltrated T regulatory cells (Tregs) and suppressed Th1 and Th17 cell-driven colon inflammation. Concentration of Galectin-3 in serum and stool samples of UC patients negatively correlated with clinical, endoscopic, and histological parameters of colitis. The cutoff serum values of Galectin-3 that allowed the discrimination of mild from moderate and moderate from severe colitis were 954 pg/mL and 580 pg/mL, respectively. Fecal levels of Galectin-3 higher than 553.44 pg/mL indicated attenuation of UC. In summing up, Galectin-3 regulates the cross-talk between colon-infiltrating DCs and Tregs and represents a new biomarker for monitoring the progression of UC.

## 1. Introduction

Ulcerative colitis (UC) is a chronic inflammatory bowel disease (IBD) confined to the colon [1]. It is characterized by epithelial cell destruction, connective tissue defects, ulceration of the mucosa and continuous or discontinuous mucosal inflammation, which results in remitting and relapsing clinical course [2]. Symptoms are often unspecific and may vary with the disease course making it difficult to clinically assess disease activity [3]. Accordingly, the standard of care implies a close monitoring of the histological and endoscopic parameters of disease severity in order to timely prevent life threatening aggravation of disease [4]. While endoscopic evaluation of UC progression is the gold standard for the follow-up of UC patients, this diagnostic approach is invasive and time-consuming [3,4]. Therefore, measurement of non-invasive inflammation-related biomarkes (C-reactive protein (CRP), fecal calprotectin (FC), and lactoferrin (FL)) in serum and stool samples of UC patients are nowadays used in clinical practice to defer the need for colonoscopic evaluation [5,6]. Keeping in mind that these biomarkers have some limitations related to alimentary ingestion, non-steroidal anti-inflammatory drugs intake and inability of distinguishing inflammation from infection, there is still a need for a new, non-invasive biomarker, which should complement CRP, FC and FL in determination of UC progression [7].

Considerable interest has recently arisen in the intriguing immunomodulatory properties of Galectin 3 (Gal-3), a member of β-galactoside-binding lectins, which regulates numerous biological processes in the gut including migration, proliferation, and activation of resident and colon-infiltrated immune cells. Concentration of Gal-3 was significantly increased in serum samples of UC patients compared to healthy controls [8]. Interestingly, lack of Gal-3 expression showed different effects on the effector function of colon-infiltrated macrophages and T cells [9,10,11]. Gal-3 is highly expressed on colonic macrophages of UC patients and its deficiency inhibits activation of NLRP3 inflammasome and production of inflammatory cytokines in these cells, resulting in attenuation of acute colitis [9]. On contrary, after the treatment with recombinant Gal-3, T cells of UC patients developed immunosuppressive phenotype and are not able to optimally proliferate [10,11,12]. Additionally, adoptive transfer of Gal-3-primed T cells significantly attenuated chronic dextran sodium sulphate sodium (DSS)-induced colitis in mice [10,11,12]. Keeping in mind that macrophages play important roles in the induction phase of colitis, while T cells are the most important effector immune cells for progression of colon inflammation, herewith, by using clinical data and complementary in vitro and in vivo approaches, we tried to delineate molecular mechanisms, which are responsible for Gal-3-dependent regulation of immune response in the inflamed gut and to determine whether Gal-3 may be used as a valuable biomarker for monitoring UC progression. We demonstrated that, during the induction phase of colitis, Gal-3 promotes interleukin (IL)-1β-induced polarization of colonic macrophages towards inflammatory M1 phenotype, while in the recovery phase of colitis Gal-3 controls T cell-driven colon inflammation by down-regulating immunosuppressive function of dendritic cells (DCs) in the gut. Gal-3 is required for optimal toll-like receptor (TLR)-4-dependent activation of indoleamine 2,3-dioxygenase-1/kynurenine (IDO-1/KYN) pathway in DCs which promotes expansion of colon-infiltrated T regulatory cells (Tregs), and therefore, suppresses Th1 and Th17 cell-driven chronic colitis. Since concentration of Gal-3 in serum and stool samples of UC patients negatively correlated with clinical, endoscopic, and histological parameters of disease severity, we proposed that Gal-3 might serve as a valuable biomarker for monitoring disease progression. Accordingly, we determined the precise “cut off” values of serum and fecal Gal-3, which might be used for distinguishing mild, moderate, and severe forms of UC.

## 2. Materials and Methods

### 2.1. Study Population

This study recruited a total of 65 patients with UC (38 male and 27 female) with a median age of 50 years (range 26–82). Additionally, 30 healthy subjects (16 male and 14 female) with a median age of 48 years (range 25–75), whose checkups were finished at the Clinical Center of Kragujevac as a routine item, were randomly chosen to represent the general population as healthy controls. Patients with UC were classified into three groups: Mild, moderate, and severe colitis. All endoscopies were performed by the same experienced endoscopist. The exclusion criteria were pregnancy, organ-specific and systemic acute or chronic inflammatory disorders, autoimmune diseases, severe, and life-treating diseases (heart failure, arrythmias, renal insufficiency, respiratory insufficiency, fulminant liver injury), and malignant disease, including colorectal cancer. Since galectin profiling could not distinguish Crohn’s disease and UC-related pathological changes in the gut [13], in order to specifically determine the role of Gal-3 in the pathogenesis of UC, patients with Crohn’s disease were excluded from the study. The study was conducted at the Center for Gastroenterology, Clinical Center of Kragujevac and Center for Molecular Medicine and Stem Cell Research, Faculty of Medical Sciences, University of Kragujevac, Serbia and was approved by the Ethics Committees of these institutions. The Principle of Good Clinical Practice and the Declaration of Helsinki were adhered to at all times. All patients gave their informed consent for blood and tissue analysis. Patients were under continuous medical supervision at the Clinical Center of Kragujevac.

### 2.2. Measurements of Gal-3, IDO Activity, Concentration of Cytokines, and Fecal Calprotectin in Serum and Fecal Samples of Patients with UC and Healthy Controls

Blood and fecal samples were prepared as previously described [14]. IDO activity was determined by spectrophotometric assay for KYN in the serum and fecal samples of patients with UC and healthy controls [15]. Gal-3, IL-10, C-X-C motif chemokine 11 (CXCL11), IL-17, and fecal calprotectin were measured in serum and/or fecal samples of UC patients and healthy subjects by using commercially available enzyme-linked immunosorbent assay (ELISA) kit sets, according to the manufacturer’s instructions [16].

### 2.3. Isolation of Colon-Infiltrating Tregs from the Colon Samples of UC Patients and Flow Cytometry Analysis of Their Phenotype and Function

The phenotype of colon-infiltrating Tregs was determined by flow cytometry [17]. Briefly, about 1 × 10^6^ cells per sample were incubated with antihuman CD4 and IL-10 antibody conjugated with fluorescein isothiocyanate (FITC; BD Biosciences, Franklin Lakes, NJ, USA), phycoerythrin (PE; BD Biosciences), peridinin chlorophyll A protein (PerCP; BD Biosciences) or allophycocyanin (APC; BD Biosciences). For the intracellular staining, cells were previously stimulated with phorbol myristate acetate (PMA) and ionomycin for 4 h at 37 °C with the addition of 1 μg/mL Golgi plug. Intracellular staining for forkhead box P3 (Foxp3) was performed using the BD Bioscience fixation/permeabilization buffer kit. Flow cytometric analysis was conducted on a BD Biosciences FACSCalibur and analyzed by using the flowing software analysis program.

### 2.4. Cytokine Production in T Cells from UC Patients

T cells (1 × 10^6^ cells per well), isolated from the population of peripheral blood mononuclear cells of UC patients, were primed with 5 μg/mL Concanavalin A (polyclonal potent activator of T cells) and cultured in Dulbecco’s Modified Eagle Medium (DMEM) supplemented with 10% fetal bovine serum (FBS), 2 mmol/L Lglutamine, 1 mmol/L penicillin-streptomycin, and 1 mmol/L mixed nonessential amino acids (Sigma, Munich, Germany) at 37 °C in 5% CO_2_ in a fully humidified atmosphere for 48 h, as previously suggested [18]. The levels of transforming growth factor beta (TGF-β), IL-10, IL-17, and interferon gamma (IFN-γ) were determined in cell culture supernatants by ELISA sets and correlated with concentrations of Gal-3.

### 2.5. Animals

All animal experiments were approved by and conducted in accordance with the guidelines of the Animal Ethics Committee of the Faculty of Medical Sciences, University of Kragujevac, Serbia. Breeding pairs of Gal-3^−/−^ and WT C57BL/6 mice of the same substrain, initially obtained from Dr. Daniel Hsu (University of California, Davis, CA, USA), were maintained in animal facilities of the Faculty of Medical Sciences, University of Kragujevac, Serbia. The total number of 10 mice per experimental group and 6 mice per control group were used. Mice used for experiments were male and age-matched (8 weeks old) and housed with a 12 h light-dark cycle and were administered by standard laboratory chow and water ad libitum.

### 2.6. Induction and Evaluation of Colitis in Mice

DSS (2.2%, molecular weight 40 kDa; TdB Consultancy, Uppsala, Sweden) was given to mice in place of normal drinking water for 5 days ad libitum. After that, DSS was substituted with normal drinking water for 3 weeks ad libitum [19]. Control mice had access to DSS-free water.

Animal weight loss, stool consistency, and fecal blood were recorded daily for each animal. These parameters were used to calculate an average Disease Activity Index (DAI) for each animal [20].

For histological analysis, colons were removed from euthanized mice, rinsed with phosphate buffer solution (PBS), and cut longitudinally before being rolled into ‘Swiss roll’ [21]. Swiss-rolled colons were fixed in formalin, embedded in paraffin, 5 μm sections were stained with haematoxylin-eosin (H&E), and examined in a blinded manner by a pathologist. Sections were analyzed for damage to epithelium, including damage to crypts, submucosal edema, hemorrhage, and infiltration by immune cells. The histology score for each mouse was calculated as the sum of two parameters (infiltration and damage of crypts) [22].

### 2.7. Flow Cytometry Analysis of Colon Infiltrating Immune Cells of DSS-Treated Mice

Isolation of immune cells from lamina propria of DSS-treated mice and flow cytometry analysis were conducted as previously described [9]. Flow cytometry followed routine procedures by using 1 × 10^6^ cells per sample, which were incubated with anti-mouse F4/80, CD4, CD11c, CD80, CD40, and CD86 conjugated with FITC, PE, PERCP, or APC (BD Biosciences, Franklin Lakes, NJ). For the intracellular staining, cells were previously stimulated with PMA and ionomycin for 4 h at 37 °C with the addition of 1 μg/mL Golgi plug. Following extracellular staining, cells were fixed, permeabilised, and stained for IL-10, Foxp3, IL-17, IFN-γ, IL-12, IL-6, IL-4, TGF-β, and IL-23 conjugated with FITC, PE, PERCP, or APC (BD Biosciences, Franklin Lakes, NJ). Flow cytometric analysis was conducted on a BD Biosciences FACSCalibur and analyzed by Flowing Software analysis program.

### 2.8. Measurements of Cytokines in Serum Samples of DSS-Treated Mice

The commercial ELISA sets (R&D Systems, Minneapolis, MN, USA) were used to determine the concentration of IL-12, IL-6, IL-17, IFN-γ, tumor necrosis factor alpha (TNF-α), TGF-β, and IL-10 in serum samples of control and DSS-treated animals [9].

### 2.9. Isolation and TLR-4-Priming of DCs

DCs were isolated from spleens of 10 WT and Gal-3^−/−^ mice by magnetic cell sorting. Single-cell suspensions of mononuclear cells were labelled with CD11c MicroBeads (Miltenyi Biotec, Bergisch Gladbach, Germany). The labelled cells were subsequently positively selected using MACS Column (Miltenyi Biotec) and MACS Separator (Miltenyi Biotec) [23]. Isolated DCs (3 × 10^5^/mL) were primed with Lipopolysaccharide (LPS, 10 ng/mL) for 48 h [24].

### 2.10. Pharmacological Inhibition of Gal-3 in DCs

Pharmacological inhibition of Gal-3 in DCs was induced by selective Gal-3 inhibitors. WT DCs were cultured in the presence of Davanat (DCs^Davanat^ 15 μg/mL) for 24 h, according to previously published protocol [25].

### 2.11. Determination of IDO1 Activity

IDO1 activity in serums samples as well as in supernatants of TLR-4-primed WTDCs, Gal-3^−/−^DCs and DCs^Davanat^ was determined by spectrophotometric measuring of KYN [14,23].

### 2.12. Adoptive Transfer of TLR-4 Primed DCs in DSS-Treated Mice

For adoptive transfer experiments, TLR-4 primed WT and Gal-3^−/−^ animals, were transferred (intraperitoneally; 2 × 10^5^ DCs/mouse) into DSS-treated WT recipients on the 5th and 15th day [26,27].

### 2.13. Co-culture of TLR-4-Primed DCs and Tregs

Tregs were isolated from the population of spleen mononuclear cells obtained from 10 WT mice by magnetic cell sorting (Tregs isolation kit, Miltenyi Biotec, Bergisch Gladbach, Germany) [28] and co-cultured in the presence of TLR-4-primed WTDCs, Gal-3^−/−^DCs, or WTDCs^Davanat^ [29]. After 48h of culture, Tregs were collected for co-culture experiments with T cells and frozen at −80 °C until gene expressions were measured by real-time PCR.

### 2.14. Co-Culture of Tregs and Effector T Cells

Tregs, previously cultured in the presence of TLR-4 primed WTDCs, Gal-3^−/−^DCs, or WTDCs^Davanat^ were co-cultured with Con-A primed T cells. After 48 h, the expression of IFN-γ, IL-17, and IL-10 in activated T cells was evaluated by real time PCR analysis.

### 2.15. RNA Isolation and Real-Time PCR Analysis

Total RNA from T cells was extracted using TRIzol reagent (Invitrogen, Carlsbad, CA, USA). Total RNA (2 μg) was reverse transcribed to cDNA using High-Capacity cDNA Reverse Transcription Kit (Applied Biosystems, Foster City, CA, USA). qRT-PCR was performed using Power SYBR MasterMix (Applied Biosystems) and miRNA-specific primers for IFN-γ, IL-17, IL-10, and β-actin as a housekeeping gene. qPCR reactions were initiated with a 10-min incubation time at 95 °C followed by 40 cycles of 95 °C for 15 s and 60 °C for 60 s in a Mastercycler ep realplex (Eppendorf, Hamburg, Germany). Relative expression of genes was calculated according to the formula 2-(Ct-Ctactin), where Ct is the cycle threshold of the gene of interest and Ctactin is the cycle threshold value of the housekeeping gene (β-actin) [30].

### 2.16. Statistics

Data were expressed as the mean ± standard error of the mean (SEM) for each group. Results were analysed by Student’s t test and Pearson’s or Spearman’s correlation coefficient. Fisher’s exact test was used to assess survival differences between groups. A receiver operated characteristic (ROC) curve was generated by plotting the false positive fraction versus the true-positive fraction for every possible cutoff score. Statistical analyses were performed using SPSS 25.0 for Windows software (SPSS Inc., Chicago, IL, USA). The difference was considered significant when *p* < 0.05.

## 3. Results

### 3.1. Gal-3 Had Pro-Inflammatory Role in Induction Phase of Colitis, but Its Deficiency Significantly Impaired Recovery from DSS-Induced Colon Injury

**Figure 1:**
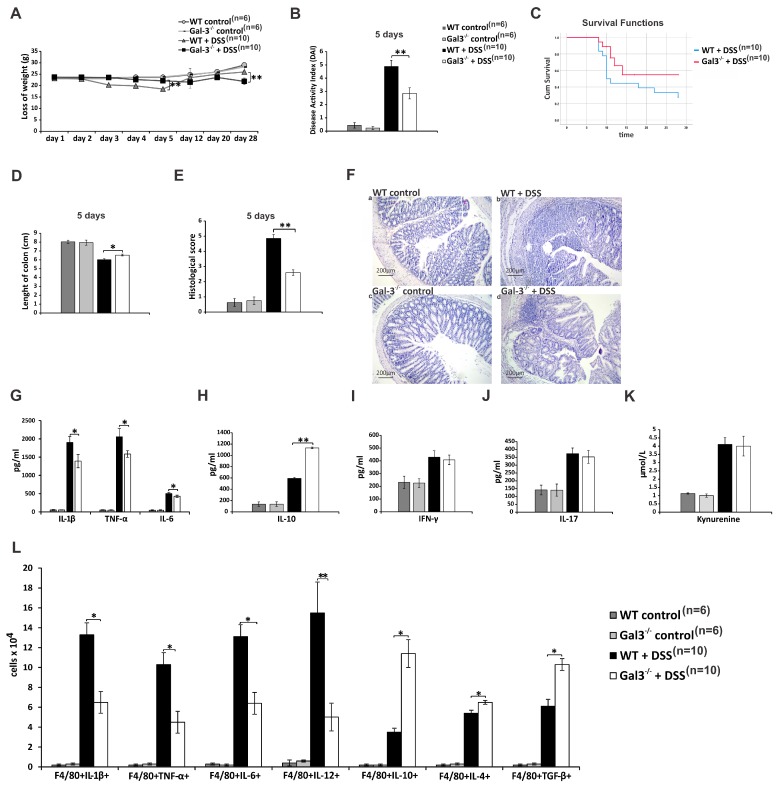
Gal-3 has pro-inflammatory role in induction phase of DSS-induced colitis. 2.2% DSS was given to mice for 5 days while regular drinking water was given to control animals. DSS-induced loss of weight (**A**); Disease Activity Index DAI (**B**); and survival rate (**C**) 5 days after colitis induction. Length of colon (**D**) and histological score (**E**) of DSS-treated mice. Haematoxylin and eosin (H&E) stained colon tissue samples of DSS-treated mice (magnification 100×) (**F**). Concentrations of IL-1β, TNF-α, IL-6 (**G**), IL-10 (**H**), IFN-γ (**I**), IL-17 (**J**), and KYN (**K**) in serum samples of DSS-treated mice at day 5. The total number M1 and M2 macrophages in colons of DSS-treated animals (**L**). Data are presented as mean ± standard error of the mean (SEM); n = 10 mice per experimental and 6 mice per control groups. * *p* < 0.05, ** *p* < 0.001.

In order to determine which molecular and cellular mechanisms were responsible for Gal-3-dependent regulation of immune response during inductive and recovery phases of colitis, we compared the differences in clinical course, survival rate, and colon architecture between WT and Gal-3^−/−^ mice during the development and progression of acute DSS-induced colitis. DSS-treated WT animals start to lose their weight few days before Gal-3^−/−^ mice (at day 3) and body weight loss was significantly lower in DSS-treated WT animals compared to Gal-3^−/−^ mice at day 5 (Figure 1A). WT and Gal-3^−/−^ mice continued to lose body weight after DSS removal. Seven days upon DSS withdrawal (at day 12), WT mice started to recover their weight, reaching their original weight at day 28. On the contrary, Gal-3^−/−^ mice continuously lose their weight resulting in significantly higher weight loss compared to their WT counterparts at day 28 (Figure 1A).

**Figure 2:**
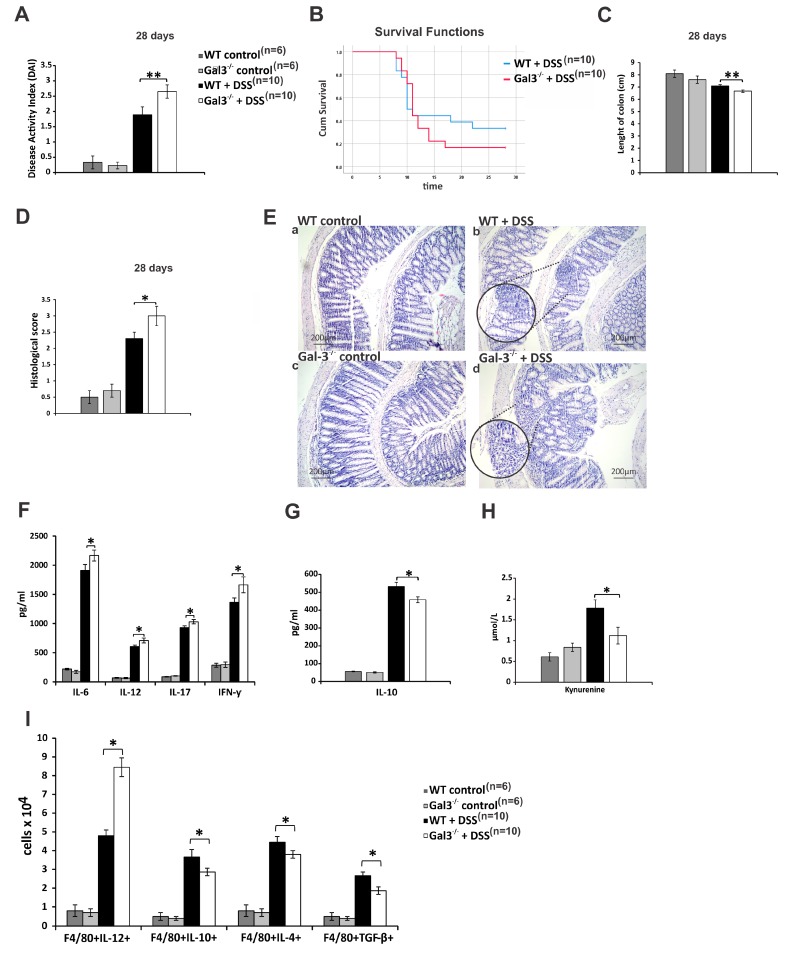
Gal-3 deficiency significantly aggravated colon injury and inflammation in chronic phase of DSS-induced colitis. DAI (**A**), survival rate (**B**), length of colon (**C**), histological score (**D**), representative H&E stained colon tissue samples (**E**), serum concentration of IL-6, IL-12, IL-17, IFN-γ (**F**), IL-10 (**G**), KYN (**H**), and total number of colon-infiltrated M1 and M2 macrophages (**I**) in DSS-treated WT and Gal-3^−/−^ mice, 28 days after initial DSS administration. Values are mean ± standard error of the mean (SEM) (n = 10 mice per experimental and 6 mice per control groups) * *p* < 0.05, ** *p* < 0.001.

During the inductive phase of acute colitis, clinical signs were more severe in WT animals (Figure 1B), while more aggravated disease was observed in DSS-treated Gal-3^−/−^ mice during the progression of the disease (Figure 2A). Fecal blood was detected in WT animals at day 2, whereas gross rectal bleeding and diarrhea were observed from day 4. These signs of colon injury started to appear in Gal-3^−/−^ mice from day 5. Visible blood in stool samples of DSS-treated WT mice could not be detected after day 16, and from day 12, the stool loss was not as prominent as during the induction phase of colitis. On the contrary, massive rectal bleeding and watery diarrhea were continuously observed in DSS-treated Gal-3^−/−^ mice until the end of experiment. Accordingly, DAI score was significantly lower in DSS-treated Gal-3^−/−^ mice at the end of induction phase of colitis (day 5, Figure 1B), but was significantly higher at day 28 (Figure 2A). In line with these findings, Gal-3 deficiency improved survival (Figure 1C) and significantly increased length of colon of DSS-treated mice (*p* < 0.05; Figure 1D) at the end of induction phase of colitis. On the contrary, remarkably reduced survival rate (Figure 2B) and significantly increased colonic shortening (*p* < 0.01, Figure 2C) were observed in Gal-3^−/−^ mice at day 28.

In line with these findings were the results obtained by histological analysis (Figure 1E–F and Figure 2D–E). Histological score was significantly lower in DSS-treated Gal-3^−/−^ mice at the end of induction phase of colitis (*p* < 0.01, Figure 1E). Histological analysis revealed that 5 days of DSS treatment caused significant epithelial cell damage, edema, ulceration, and extensive crypt drop-out in colons of WT mice (Figure 1Fb), while only mild evidence of crypt distortion and widening were observed in colon samples of Gal-3^−/−^ animals (Figure 1Fd). Opposite findings were observed at day 28 resulting in significantly higher histological score in DSS-treated Gal-3^−/−^ mice (*p* < 0.05; Figure 2D). Histological analyses showed that DSS-caused pathological changes (loss of intestinal epithelial cells, decreased number of crypts, and submucosal edema) were still present in the colons of WT mice, 4 weeks after initial administration of DSS (Figure 2Eb). Importantly, all of these abnormalities in colon architecture were more prominent in Gal-3^−/−^ mice (Figure 2Ed). Destruction of the entire epithelium, more prominent loss of crypts and goblet cells, accompanied with more severe submucosal edema and massive accumulation of lymphocytes were observed in the colons of DSS-treated Gal-3^−/−^ mice at day 28 (Figure 2Fd).

Genetic deletion of Gal-3 affected cytokine networking of innate immune cells in the induction phase of colitis (Figure 1G,H) and altered cytokine release from adaptive immune cells during the progression of DSS-induced colitis (Figure 2F–H). Significantly lower concentrations of inflammatory cytokines of innate immunity (*p* < 0.05 for IL-1β, TNF-α, and IL-6) were observed in serum samples of DSS-treated Gal-3^−/−^ mice at the end of induction phase of colitis (Figure 1G). Additionally, Gal-3 deficient mice had elevated serum levels of anti-inflammatory IL-10 (*p* < 0.01; Figure 1H), while there was no significant difference in serum levels of inflammatory, T cell-derived cytokines (IFN-γ and IL-17; Figure 1I,J) and immunosuppressive KYN (Figure 1K) between DSS-treated WT and Gal-3^−/−^ mice at day 5. On the contrary, serum levels of immunosuppressive IL-10 (*p* < 0.05; Figure 2G) and KYN (*p* < 0.05; Figure 2H) were significantly lower, while concentration of inflammatory Th1 cell-derived IFN-γ Th17 cell-derived IL-17 were significantly higher (*p* < 0.05; Figure 2F) in serum samples of DSS-treated Gal-3^−/−^ mice at day 28.

Macrophages orchestrate the cross-talk between colon-infiltrating immune cells in induction phase of acute DSS- colitis [31,32]. Cellular make-up of the DSS-injured colons revealed significantly lower number of inflammatory, IL-1β- (*p* < 0.05), TNF-α-( *p* <0.05), IL-6 (*p* < 0.05), and IL-12-producing M1 macrophages (*p* < 0.01), but a significantly higher number of IL-10-, IL-4, and TGF-β-producing M2 macrophages (*p* < 0.05) in the colons of DSS-treated Gal-3^−/−^ mice at day 5 (Figure 1L). On the contrary, total number of IL-10-, IL-4 and TGF-β-producing M2 macrophages was remarkably lower (*p* < 0.05) and total number of IL-12-producing M1 macrophages (*p* < 0.05) was significantly higher in the colons of DSS-treated Gal-3^−/−^ mice 28 days after the initial administration of DSS (Figure 2I).

### 3.2. Gal-3 Deficiency Resulted in Enhanced DC-Dependent Activation of Inflammatory Th1 and Th17 Cells in DSS-Induced Colitis

**Figure 3:**
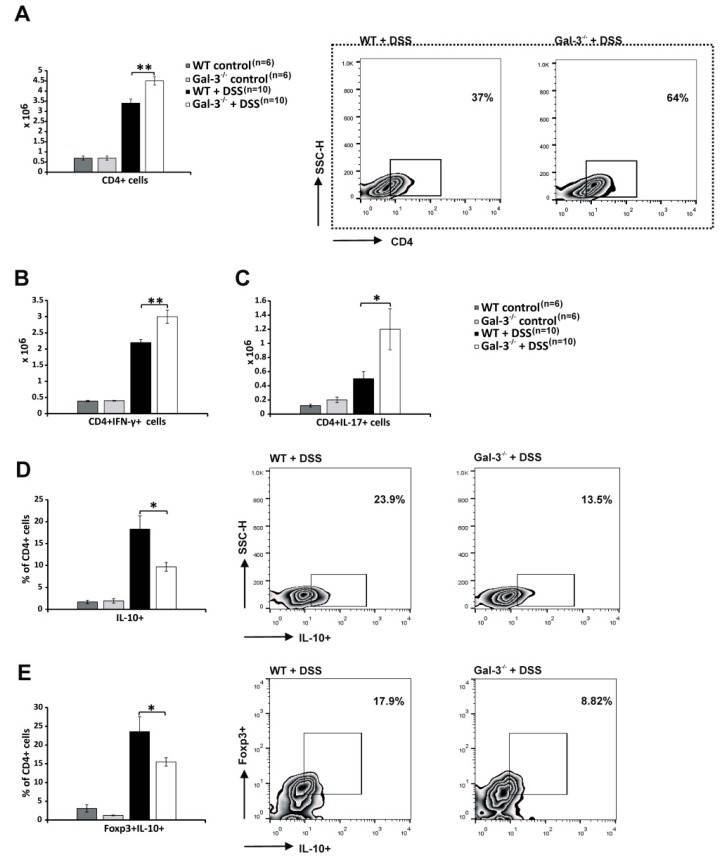
Genetic deletion of Gal-3 markedly enhanced presence of Th1 and Th17 cells in colons of DSS-treated mice. The total number of T cells in colons of DSS-treated WT and Gal-3^−/−^ mice 28 days after initial DSS administration (**A**). The total number of CD4+IFN-γ+ Th1 T cells (**B**), CD4+IL-17+ Th17 cells (**C**), percentage of regulatory T cells (CD4+IL-10+ and CD4+Foxp3+IL-10+) (**D**,**E**) with representative flow cytometry dot plots are presented. Values are mean ± standard error of the mean (SEM) (n = 10 mice per experimental and 6 mice per control groups). * *p* < 0.05, ** *p* < 0.001.

T cells have crucially important role in the progression of DSS-induced colon inflammation [33]. Accordingly, significantly higher number of CD4+ T cells was seen in colons of Gal-3^−/−^ mice 28 days after initial administration of DSS (*p* < 0.01; Figure 3A). Intracellular staining revealed significantly higher presence of colon-infiltrating, IFN-γ producing CD4+ Th1 cells (*p* < 0.01; Figure 3B) and IL-17-producing CD4+Th17 cells (*p* < 0.05; Figure 3C) in DSS-treated Gal-3^−/−^ mice. In contrast, significantly lower presence of immunosuppressive, IL-10-producing CD4+T cells (*p* < 0.05; Figure 3D) and FoxP3+CD4+Tregs (*p* < 0.05, Figure 3E) were observed in colons of DSS-treated Gal-3^−/−^ mice, suggesting that Gal-3 deficiency altered the polarization of colon-infiltrated T cells towards pro-inflammatory Th1/Th17 phenotype.

**Figure 4:**
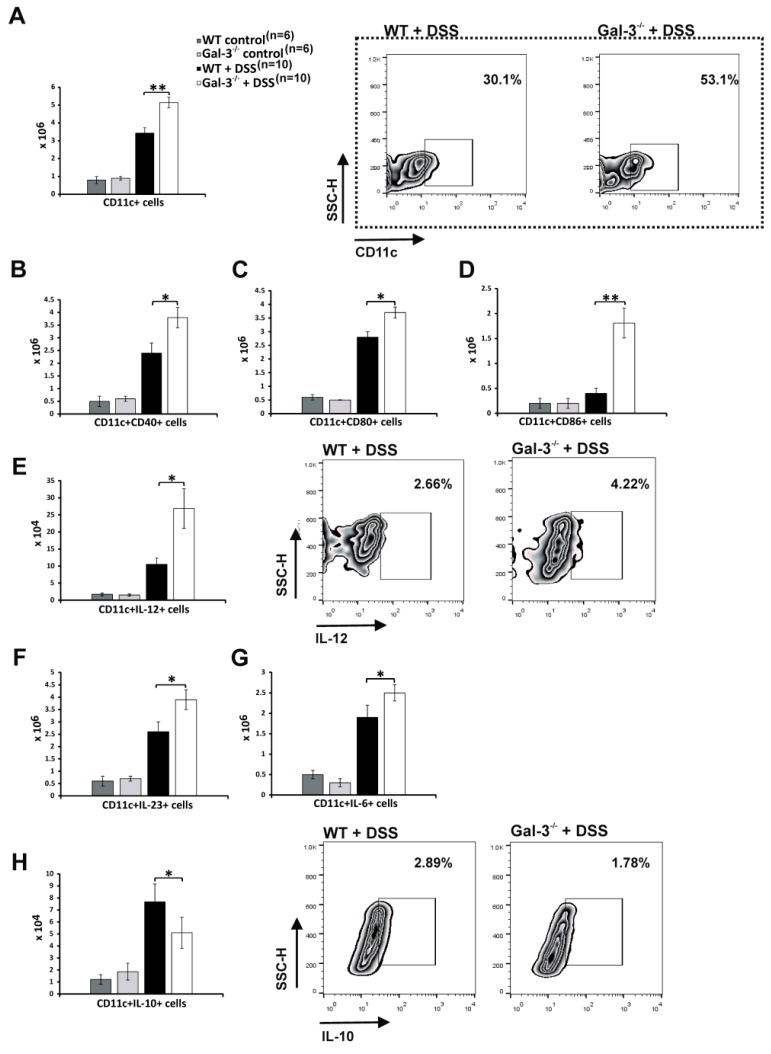
Gal-3 deletion favored development of inflammatory phenotype in colon-infiltrated DCs. The total number of CD11c+ DCs in colons of DSS-treated WT and Gal-3^−/−^ mice 28 days after initial DSS administration (**A**). The total number of CD40, CD80 and CD86-expressing CD11c+DCs (**B**–**D**) and IL-12, IL-23, IL-6, and IL-10 producing CD11c+DCs in colons of DSS-treated WT and Gal-3^−/−^ mice (**E**–**H**). Data are presented as mean ± standard error of the mean (SEM); n = 10 mice per experimental and 6 mice per control groups. * *p* < 0.05, ** *p* < 0.001.

Since DCs regulate progression of DSS-induced colitis by inducing polarization of CD4+T cells in inflammatory (Th1/Th17) or immunosuppressive (Tregs) cells [17,23], we analyzed whether genetic deletion of Gal-3 affected phenotype and function of colon-infiltrating DCs 28 days after the initial administration of DSS. Firstly, we observed a significantly higher number of CD11c+DCs in the colons of DSS-treated Gal-3^−/−^ mice (Figure 4A). Additionally, a remarkably higher number of CD40−(*p* < 0.05; Figure 4B), CD80−(*p* < 0.05; Figure 4C), and CD86−(*p* < 0.01; Figure 4D) expressing DCs were seen in the colons of DSS-treated Gal-3^−/−^ mice, suggesting that significantly increased number of activated DCs with potent antigen presenting function infiltrated DSS-injured colons of Gal-3^−/−^ animals. Intracellular staining revealed that IL-12-producing DCs (*p* < 0.05, Figure 4E), which induced generation of Th1 cells [16], as well as IL-23 and IL-6-producing DCs (*p* < 0.05, Figure 4F,G), which promoted generation of Th17 cells [17,23], were present in significantly higher number in colons of DSS-treated Gal-3^−/−^ mice. In contrast, significantly lower number of regulatory, IL-10-producing DCs (*p* < 0.05; Figure 4H), which suppressed T cell-driven colon inflammation [16], was observed in Gal-3^−/−^ animals 28 days after initial DSS administration.

### 3.3. Gal-3 is Required for Optimal TLR-4-Dependent Production of KYN in DCs

**Figure 5:**
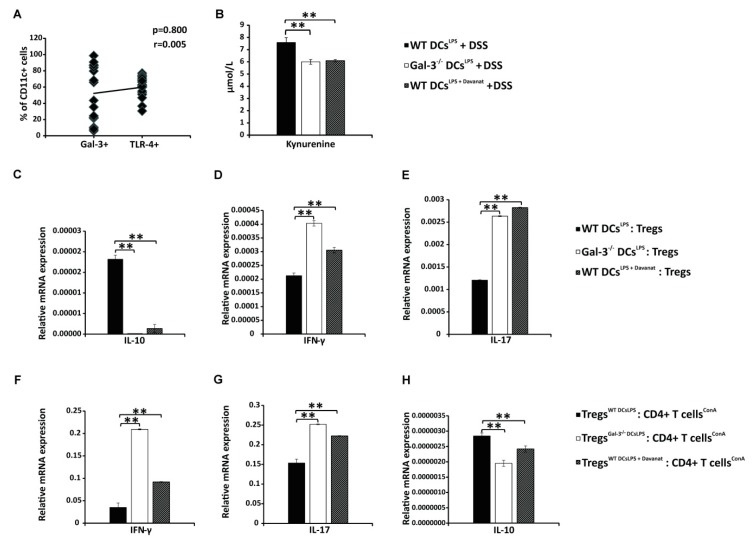
Gal-3 is required for optimal TLR-4-dependent production of KYN in DCs. Correlation between expression of Gal-3 and TLR-4 on colon-infiltrated CD11c+DCs 28 days after initial DSS administration (**A**). KYN production in LPS-primed DCs (**B**). Expression of IL-10, IFN-γ and IL-17 in Tregs which were co-cultured with LPS-primed WTDCs, Gal-3^−/−^DCs or WTDC^Davanat^ (**C**–**E**). Expression of IL-10, IFN-γ and IL-17 in activated T cells which were co-cultured with Tregs primed by LPS-stimulated WTDCs, Gal-3^−/−^DCs, or WTDC^Davanat^ (**F**–**H**). Data presented as mean ± standard error of the mean (SEM); n = 10 mice per experimental groups. ** *p* < 0.001.

It is well known that LPS priming induces activation of IDO-1/KYN pathway in DCs [34] and that DCs promote expansion of colon-infiltrating Tregs in IDO-1/KYN-dependent manner [35]. Since we noticed strong positive correlation between expression of TLR-4 and Gal-3 on colon-infiltrated DCs of DSS-treated animals (Figure 5A, r = 0.800, *p* = 0.005), we evaluated the importance of Gal-3 for TLR-4-dependent activation of IDO-1/KYN pathway in DCs. As it is shown in Figure 5B, activation of TLR-4 significantly enhanced production of immunosuppressive KYN in WT DCs (Figure 5B; *p* < 0.01). Importantly, genetic deletion as well as pharmacological inhibition of Gal-3, remarkably reduced TLR-4-dependent secretion of KYN in DCs (Figure 5B; *p* < 0.01). Significantly lower concentrations of KYN were measured in supernatants of Gal-3^−/−^ DCs^LPS^ and WT DCs^LPS+Davanat^ compared to WT DCs^LPS^ (Figure 5B, *p* < 0.01).

### 3.4. Gal-3 Deficiency Completely Abrogated Capacity of TLR-4 Primed DCs to Maintain Immunosuppressive Function of Tregs

Genetic deletion as well as pharmacological inhibition of Gal-3 in WT DCs^LPS^ diminished their capacity to maintain immunosuppressive phenotype of Tregs (Figure 5C) and to prevent their trans-differentiation in inflammatory Th1 or Th17 cells (Figure 5D,E). Decreased expression of anti-inflammatory IL-10 (Figure 5C; *p* < 0.01) and increased expression of inflammatory IFN-γ (Figure 5D; *p* < 0.01) and IL-17 (Figure 5E; *p* < 0.01) were noticed in Tregs, which had been cultured with Gal-3^−/−^ DCs^LPS^ or WT DCs^LPS+Davanat^ compared to Tregs that were cultured with WT DCs^LPS^.

Additionally, Tregs generated by Gal-3^−/−^ DCs^LPS^ or WT DCs^LPS+Davanat^ were not able to optimally suppress production of IFN-γ (Figure 5F; *p* < 0.01) and IL-17 (Figure 5G; *p* < 0.01) in activated CD4+ T cells, indicating the importance of Gal-3 for DC-driven regulation of cross-talk between Tregs and effector T cells.

### 3.5. Genetic Deletion of Gal-3 Diminished Capacity of TLR-4-Primed DCs to Attenuate DSS-Induced Colitis

**Figure 6:**
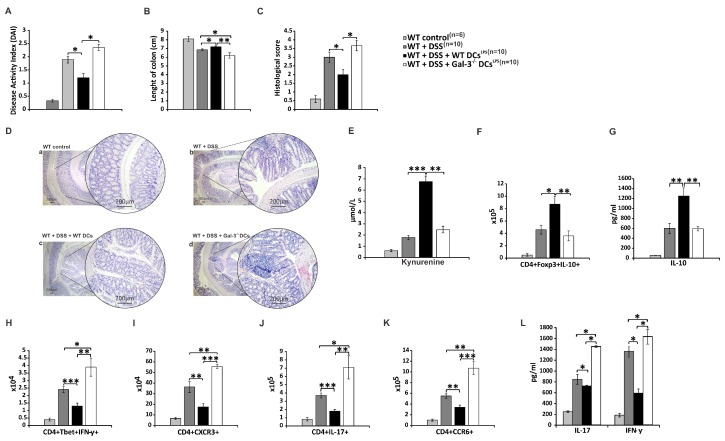
Genetic deletion of Gal-3 diminished capacity of TLR-4-primed DCs for attenuation of chronic colitis. DAI (**A**), length of colon (**B**), histological score (**C**), representative H&E (**D**), serum levels of KYN (**E**) and IL-10 (**G**), total number of colon-infiltrated immunosuppressive CD4+Foxp3+IL-10+ Tregs (**F**), serum level of IL-10 (**G**), total number of colon-infiltrated inflammatory CD4+T-bet+IFN-γ+Th1 cells (**H**), CD4+CXCR3+Th1 cells (**I**), CD4+IL-17+Th17 cells (**J**) and CD4+CCR6+Th17 cells (**K**), serum concentration of IFN-γ and IL-17 (**L**), and 28 days after adoptive transfer of LPS-primed WTDCs or Gal-3^−/−^DCs in DSS-treated mice. Values are mean ± standard error of the mean (SEM) (n = 10 mice per experimental groups and 6 mice per control group). * *p* < 0.05, ** *p* < 0.01; *** *p* < 0.001.

In order to demonstrate crucial role of Gal-3 for DCs^LPS^-dependent expansion of Tregs and consequent attenuation of DSS-induced colitis, we injected WT DCs^LPS^ or Gal-3^−/−^ DCs^LPS^ in DSS-treated mice. Transfer of WT DCs^LPS^ promoted recovery from DSS-induced colitis (Figure 6A–D). Significantly lower DAI score (*p* < 0.05; Figure 6A) (evidenced by improved stool consistency and diminished rectal bleeding), increased colon length (*p* < 0.05; Figure 6B) and reduced histology score (*p* < 0.05; Figure 6C) were observed in DSS-treated mice that received WT DCs^LPS^. Histological analysis of their colon sections revealed reduced number of mucosal erosions and ulcerations, lower hyperplasia, and decreased infiltration of lymphocytes (Figure 6Dc). Importantly, adoptive transfer of WT DCs^LPS^ significantly increased serum level of KYN in DSS-treated mice (*p* < 0.001; Figure 6E), which resulted in the expansion of colon-infiltrating IL-10-producing Tregs (*p* < 0.05; Figure 6F) and elevated serum level of IL-10 (*p* < 0.01; Figure 6G). On contrast, significantly lower number of IFN-γ-producing, T-bet and CXCR3-expressing Th1 cells (*p* < 0.01; Figure 6H,I), and IL-17-producing and CCR6-expressing Th17 cells (*p* < 0.01; Figure 6J,K), accompanied by significantly reduced serum levels of IFN-γ and IL-17 (*p* < 0.05; Figure 6L), were observed in DSS-treated mice after adoptive transfer of WT DCs^LPS^, indicating that WT DCs^LPS^ alleviated DSS-induced colitis by suppressing Th1 and Th17 cell-driven inflammatory response in the gut.

Importantly, the adoptive transfer of Gal-3^−/−^ DCs^LPS^ did not alter serum level of KYN and total number of colon-infiltrating Tregs in DSS-treated mice (Figure 6E,F), suggesting that Gal-3 was required for the optimal production of KYN in TLR-4-primed DCs and for consequent Treg-based alleviation of Th1/Th17 cell-driven colitis.

### 3.6. Serum Gal-3 May Serve as A Valuable Marker for Monitoring the Progression of UC

**Figure 7:**
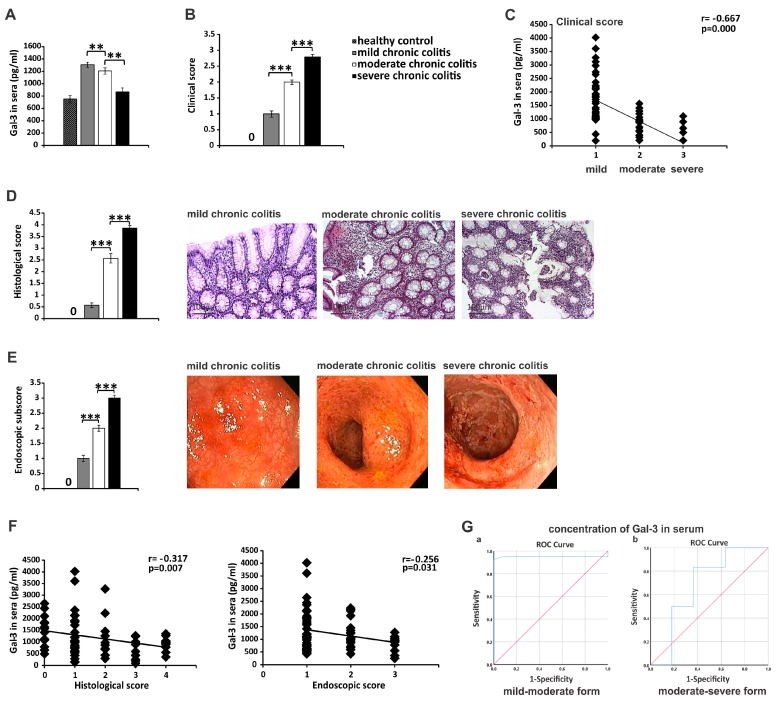
Serum Gal-3 may serve as a valuable marker for monitoring the progression of UC. The serum levels of Gal-3 (**A**), clinical score (**B**), histological score (**D**), endoscopic score (**E**) in UC patients with mild, moderate or severe forms of UC. Correlation between clinical score and serum levels of Gal-3 (**C**). Representative histological sections (**D**, magnification of 200×) and endoscopic images (**E**) of UC patients with mild, moderate, or severe forms of UC. (**D**). Correlation between serum levels of Gal-3 and histological or endoscopic score (**F**). ROC curve illustrate the specificity and sensitivity of Gal-3 serum concentration, comparing mild chronic colitis with moderate chronic colitis (left panel) and specificity and sensitivity of sera Gal-3, comparing moderate chronic colitis with severe chronic colitis (right panel) (**G**). Mean ± standard error of the mean; ** *p* < 0.01; *** *p* < 0.001.

In order to investigate the relevance of experimental findings for corresponding human pathology, we analyzed the concentration of Gal-3 in serum samples of healthy controls and in patients with UC who were divided into three groups based on the disease severity (UC patients with mild, moderate and severe colitis) (Table 1). The serum level of Gal-3 was higher in UC patients compared with healthy controls (Figure 7A). Interestingly, a significantly higher serum levels of Gal-3 were observed in patients with mild UC than in patients with moderate UC and in patients with moderate UC than in patients with severe UC (*p* < 0.01; Figure 7A). The clinical score negatively correlated with serum concentrations of Gal-3 (r = −0.667, *p* < 0.0005; Figure 7C). These findings were in line with differences in histological (*p* < 0.001; Figure 7D) and endoscopic scores (*p* < 0.001; Figure 7E) between these three groups of UC. In line with these results, serum levels of Gal-3 negatively correlated with histological score (r = −0.317, *p* < 0.01; Figure 7F left panel) and with endoscopic subscore (r = −0.256, *p* < 0.05; Figure 7F right panel).

ROC analysis was performed to evaluate the usefulness of serum Gal-3 levels for classification of UC patients with mild, moderate or severe colitis. ROC analysis showed that serum Gal-3 can be used a valuable marker for monitoring UC progression (sensitivity 95%, specificity 91.7%; Figure 7G). The cutoff serum values of Gal-3 that allowed the discrimination of mild from moderate and moderate from severe UC were 954pg/mL and 580pg/mL, respectively.

### 3.7. Increased Serum Levels of Gal-3 Indicates Overproduction of Immunosuppressive Cytokine in UC Patients

**Figure 8:**
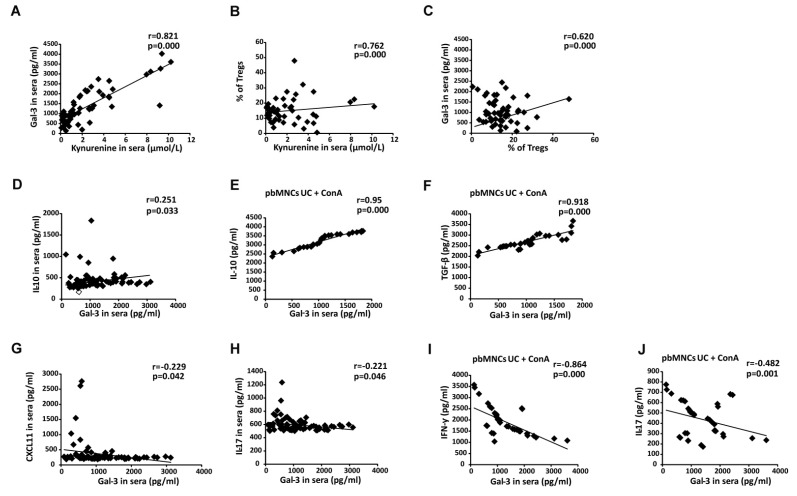
Increased serum levels of Gal-3 indicates overproduction of immunosuppressive cytokines in UC patients. Correlation between serum concentrations of Gal-3 and KYN (**A**), KYN and percentage of colon-infiltrated Tregs (**B**), serum levels of Gal-3 and percentage of colon-infiltrated Tregs (**C**), serum concentration of Gal-3 and IL-10 (**D**). Correlation between serum level of Gal-3 and concentration of IL-10 (**E**) and TGF-β (**F**) in supernatants of Con A-stimulated pbMNCs. Correlation between serum levels of Gal-3 and CXCL11 (**G**) and between serum concentration of Gal-3 and IL-17 (**H**). Correlation between serum level of Gal-3 and concentration of IFN-γ (**I**) and IL-17 (**J**) in supernatants of Con A-stimulated pbMNCs.

Serum concentration of Gal-3 was in a strong positive correlation with serum levels of KYN (r = 0.821, *p* < 0.0005; Figure 8A). Since KYN is mainly responsible for expansion of immunosuppressive Tregs [16], there was a strong positive correlation between percentage of colon-infiltrating Tregs and serum level of KYN (r = 0.762, *p* < 0.0005; Figure 8B) in UC patients. Importantly, serum levels of Gal-3 positively correlated with the percentage of colon-infiltrating Tregs (r = 0.620, *p* < 0.0005, Figure 8C) and with the serum levels of immunosuppressive IL-10 (r = 0.251, *p* = 0.033, Figure 8D) in UC patients. In line with these findings, there was strong positive correlation between serum concentration of Gal-3 and capacity of pbMNCs to produce immunosuppressive cytokines (IL-10 (r = 0.950, *p* < 0.0005, Figure 8E) and TGF-β (r = 0.918, *p* < 0.0005, Figure 8F)). In contrast, there was a negative correlation between serum levels of Gal-3 and inflammatory Th1-related chemokine CXCL11 (r = –0.229, *p* = 0.042, Figure 8G) and IL-17 (r = –0.221, *p* = 0.046, Figure 8H). In an analogy, serum concentration of Gal-3 negatively correlated with the capacity of pbMNCs to produce IFN-γ (r = –0.864, *p* < 0.0005, Figure 8I) and IL-17 (r = –0.482, *p* = 0.001, Figure 8J).

### 3.8. Measurement of Fecal Gal-3 May Be Used for Assessing Severity of UC

**Figure 9:**
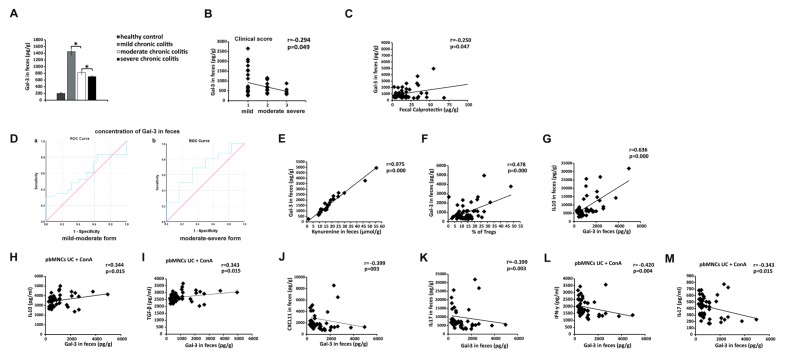
Fecal Gal-3 as biomarker for monitoring the progression of UC. Fecal concentration of Gal-3 (**A**). Correlation between fecal levels of Gal-3 and clinical score (**B**) and fecal calprotectin (**C**). ROC curve illustrating the specificity and sensitivity of Gal-3 fecal concentration, comparing mild chronic colitis with moderate chronic colitis (left panel) and specificity and sensitivity of fecal Gal-3, comparing moderate chronic colitis with severe chronic colitis (right panel) (**D**). Correlation between fecal concentration of Gal-3 and KYN (**E**), percentage of colon-infiltrated Tregs (**F**), fecal levels of IL-10 (**G**). Correlation between fecal level of Gal-3 and concentration of IL-10 (**H**) and TGF-β (**I**) in supernatants of Con A-stimulated pbMNCs. Concentration between fecal levels of Gal-3 and CXCL11 (**J**) and IL-17 (**K**). Correlation between fecal level of Gal-3 and concentration of IFN-γ (**L**) and IL-17 (**M**) in supernatants of Con A-stimulated pbMNCs. Mean ± standard error of the mean; * *p* < 0.05.

In a similar manner that was observed in serum samples (Figure 7), the concentration of Gal-3 in stool samples of UC patients was remarkably higher than in healthy controls (Figure 9A). Importantly, fecal level of Gal-3 was significantly higher in patients with mild UC than in patients with moderate UC and severe UC (*p* < 0.05; Figure 9A) and it negatively correlated with disease progression (r = −0.294, *p* = 0.049, Figure 9B). Importantly, we noticed negative correlation between fecal levels of Gal-3 and concentration of FC (r = −0.250, *p* = 0.047, Figure 9C), used as a reliable biomarker in assessing disease progression in UC patients [36,37,38]. ROC analysis revealed that, in addition to FC, fecal Gal-3 might also be used as a biomarker for monitoring severity of UC (Figure 9D). Concentration of fecal Gal-3 higher than 553.44 pg/mL indicates attenuation of UC with sensitivity of 72.7% and specificity 60.9%.

In a similar manner that was observed in serum samples (Figure 7), an increased fecal level of Gal-3 indicates overproduction of immunosuppressive cytokines. There was strong positive correlation between fecal levels of Gal-3 and fecal concentration of immunosuppressive KYN (r = 0.975, *p* < 0.0005, Figure 9E). An increased fecal level of Gal-3 also correlated with a higher presence of immunosuppressive colon-infiltrating Tregs (r = 0.478, *p* < 0.0005, Figure 9F) and with elevated concentration of anti-inflammatory IL-10 in stool samples (r = 0.636, *p* < 0.0005, Figure 9G). In line with these findings, there was positive correlation between fecal levels of Gal-3 and capacity of pbMNCs to produce immunosuppressive cytokines (IL-10 (r = 0.344, *p* = 0.015, Figure 9H) and TGF-β (r = 0.343, *p* = 0.015, Figure 9I)). On the contrary, fecal Gal-3 negatively correlated with fecal levels of Th1/Th17-related inflammatory mediators (CXCL11 (r = −0.399, *p* = 0.003, Figure 9J) and IL-17 ((r = −0.399, *p* < 0.0005, Figure 9K)) and with a capacity of pbMNCs to produce IFN-γ (r = −0.420, *p* = 0.004, Figure 9L) and IL-17 (r = −0.343, *p* = 0.015, Figure 9M).

## 4. Discussion

Herewith, by using an experimental model of DSS-induced colitis and clinical data, we demonstrated important protective role of Gal-3 in attenuation of UC. Our results indicated that Gal-3 is required for TLR-4-dependent activation of IDO-1/KYN pathway in colon-infiltrating DCs and for consequent Tregs-based suppression of Th1/Th17 cell-driven colon inflammation. Furthermore, we proposed that the measurement of serum and fecal levels of Gal-3 in UC patients might represent a valuable diagnostic tool for monitoring disease progression.

Different subpopulations of immune cells play distinct roles in the induction and progression of DSS-induced colitis [32]. Macrophages play crucially important role in the acute phase of DSS-induced colitis while T cells orchestrate colon-infiltrated immune cells during the progression of DSS-caused colon inflammation [31,32]. During the onset of DSS-induced colitis, CD14/TLR4:LPS-dependent interaction between macrophages and bacteria, which have passed through DSS-injured colonic epithelium, results in the activation of NLRP3 inflammasome, which leads to the enhanced production of IL-1β in colonic macrophages [39]. Several lines of evidence suggested that IL-1β-dependent activation of IL-1 receptor (IL-1R) in gut-infiltrating immune cells have crucially important role in the development of colon inflammation during the onset of DSS-induced colitis [40,41,42,43,44]. When LPS-activated, inflammatory M1 macrophages produce large amounts of IL-1β, it binds to IL-1R and induces increased secretion of pro-inflammatory cytokines (TNF-α, IL-6, IL-12, and IL-18) and chemokines (CXCL11 and CCL20) in neighboring macrophages, neutrophils and DCs resulting in the enhanced influx of circulating CXCR3 and CCR6-expressing effector Th1 and Th17 lymphocytes in the DSS-injured colons [40,41]. Furthermore, IL-1β:IL-1R signaling activates IL-1β converting enzyme (ICE) in colon macrophages and promotes de novo synthesis and release of IL-1β, resulting in the creation of “inflammatory cytokine loop” in DSS-injured colons, which finally results in the development of severe acute colitis [42]. On the contrary, decreased production of IL-1β in LPS-activated colon macrophages and consequent low concentration of IL-1β results in sub-optimal activation of IL-1R/MyD88 signaling pathway in macrophages, neutrophils, and DCs, but facilitate the production of granulocyte-macrophage colony-stimulating factor (GM-CSF) in type 3 innate lymphoid cells (ILC3) [43]. Accordingly, low levels of IL-1β and elevated concentration of GM-CSF induce generation of immunosuppressive, M2 phenotype in colon-infiltrated macrophages during the induction phase of DSS-induced inflammation [43]. While IL-1β regulates the onset of DSS-induced colitis, neutralization of IL-1β:IL-1R signaling in colon-infiltrating macrophages failed to show beneficial effects in mice with chronic DSS-induced colitis, indicating that other immunomodulatory molecules regulate macrophage polarization and function during the progression of colon inflammation [44].

In line with these findings are our results that showed that Gal-3 was required for optimal NLRP3-dependent production of IL-1β in colonic macrophages during the development of acute colitis [9]. In an analogy, herewith we showed that during the induction phase of DSS-induced colitis Gal-3 deficient macrophages were not able to optimally produce IL-1β upon TLR4:LPS stimulation which led to their polarization towards immunosuppressive M2 phenotype (Figure 1L). However, completely opposite findings were observed during the progression of DSS-induced colon inflammation. Significantly lower number of IL-10-, IL-4- and TGF-β-producing M2 macrophages infiltrated colons of DSS-treated Gal-3^−/−^ animals 28 days after initial administration of DSS, which resulted in aggravation of DSS-induced colitis (Figure 2I). It is well known that soluble Gal-3 is required for the maintenance of M2 macrophage subpopulation in inflamed tissues [45]. M2 macrophages secrete Gal-3, which promotes alternative activation of neighboring macrophages and creates “autocrine loop” that results in alleviation of on-going inflammation [45]. Accordingly, we assume that during the induction phase of colitis, total number of M2 macrophages was higher in colons of Gal-3^−/−^ mice due to the reduced activation of NLRP3/IL-1β pathway. However, during the progression of colitis, due to the deficiency of soluble Gal-3, polarization of colonic macrophages in M2 phenotype was prevented and pool of alternatively activated macrophages in the colons of DSS-treated Gal-3^−/−^ mice could not be maintained.

Alternatively, activated M2 macrophages suppress on-going colon inflammation either directly, through the production of anti-inflammatory TGF-β and IL-10, or indirectly, by promoting expansion of immunosuppressive Tregs in the DSS-injured colons [46]. Accordingly, lower number of TGF-β and IL-10-producing colonic M2 macrophages in the colons of Gal-3^−/−^ mice at day 28 (Figure 2I) corresponded to the reduced presence of colon-infiltrated Tregs (Figure 3D-E). Tregs are considered as the main immunosuppressive cells in UC due to their capacity to suppress detrimental Th1 and Th17 cell-driven colon inflammation [47]. Th1 cells, through the production of IFN-γ, reinforce the pro-inflammatory phenotype in colonic macrophages, whereas Th17 cells, in a IL-17-dependent manner, promote activation of colon-infiltrated neutrophils, contributing to crypt abscess formation and aggravation of UC [46]. In line with these findings, aggravated colitis observed in Gal-3^−/−^ mice four weeks after DSS administration was characterized by the increased presence of colon-infiltrating Th1 and Th17 cells (Figure 3B,C).

T cell driven inflammation in the gut and polarization of naïve T cells in Tregs or in inflammatory Th1/Th17 cells is regulated by cytokine production of colon-infiltrating DCs [48]. Cellular make-up of the colons revealed significantly higher number of CD40, CD80, and CD86-expressing DCs which produce Th1 (IL-12) and Th17-related (IL-6, IL-23) cytokines (Figure 4B-G). In contrast, genetic deletion of Gal-3 resulted in the significantly lower number of regulatory, IL-10 and KYN-producing DCs (Figure 4H and Figure 5B).

Several recently published experimental and clinical studies indicated important protective role of tryptophan (TRP) and its metabolite KYN in attenuation of colon inflammation [49,50,51,52]. Administration of TRP significantly alleviated DSS-induced colon injury in mice while removing TRP from the diet remarkably increased susceptibility to experimental colitis [51,52]. These findings have been recently confirmed in clinical settings by Nikolaus and colleagues who observed significantly lower serum levels of TRP in UC patients compared to the healthy controls [49].

IDO-1/KYN pathway comprises most of TRP metabolism in the inflamed gut [53]. Accordingly, IDO-1-expressing cells regulate TRP-dependent effects in the gastrointestinal tract, including the control of host-microbiota interactions, regeneration of DSS-injured epithelial cells, and alleviation of detrimental immune response in the gut [54,55]. High expression of IDO-1 was noticed in the epithelial cells flanking ulcers or bordering crypt abscesses within the inflamed mucosa of UC patients [56]. The highest expression of IDO-1 was observed at the margin of mucosal erosions and in the reparative ulcer-associated cell lineage suggesting important role of IDO-1 in mucosal healing and regeneration of injured epithelial cells [56]. Additionally, an enhanced IDO-1 activity has been noticed in endothelial cells, fibroblasts, as well as immune cells with immunosuppressive and anti-inflammatory properties including CD8α^+^CD16^+^, CD8α^+^CD56^+^, CD8α^+^CD80^+^, CD8α^+^CD123^+^ large granular cells, and CD123+plasmacytoid DCs [57]. Through the increased IDO-1 activity colon-infiltrated immunosuppressive cells maintains immune tolerance and attenuates on-going inflammation in the gut. Decreased number of IDO-1-expressing DCs resulted in tolerance loss and aggravation of colon injury and inflammation in UC patients [57].

Several lines of evidence indicated that the cross-talk between IDO-1-expressing DCs and Tregs was crucially important for the maintenance of immune tolerance and regeneration of injured epithelial cells in the gut [17,35,54]. Colon-infiltrated regulatory DCs, in IDO-1/KYN-dependent manner, promote expression of Treg lineage-defining transcription factor FoxP3 in naive CD4+T cells enabling generation of immunosuppressive CD4+FoxP3+Tregs [35]. During TCR-mediated activation of resting Tregs, signals via the protein kinase B (PKB/Akt) and mammalian target of rapamycin (mTOR) pathways can destabilize the immunoregulatory phenotype of Tregs and cause their reprogramming into a pro-inflammatory (“ex-Tregs”) phenotype, characterized by enhanced production of IFN-γ and IL-17 [54,58,59]. Importantly, colon-infiltrating regulatory DCs may prevent transdifferentiation of Tregs in Th1 or Th17 cells [54,58,59]. Through the increased IDO-1 activity, regulatory DCs reduce concentration of TRP in the inflamed microenvironment of the gut. The low level of TRP, in turn, activates general control nonderepressible 2 (GCN2) kinase, which inhibits Akt/mTORC2 signaling in Tregs and prevents their transdifferentiation in Th1 or Th17 cells [54,60]. In line with these findings, herewith, we demonstrated that Gal-3 is required for IDO-1/KYN-dependent cross-talk between DCs and Tregs in the inflamed gut as well as for IDO-1/KYN-mediated maintainance of Treg phenotype and function. Genetic deletion of Gal-3 significantly reduced capacity of regulatory DCs to produce KYN (Figure 5B), which resulted in the reduced presence of colon-infiltrating Tregs in DSS-treated Gal-3^−/−^ mice (Figure 3E).

There was strong positive correlation between the expression of Gal-3 and expression of TLR-4 in colon-infiltrating DCs (Figure 5A). Interestingly, TRL-4 priming, significantly increased KYN production in DCs (Figure 5B). It is well known that LPS-induced activation of TLR-4 induces activation of IDO-1 and results in enhanced production of immunosuppressive KYN in regulatory DCs [34]. Since Gal-3 acts as a ligand for TLR-4 that enables sustained TLR-4 signaling [61], genetic deletion, as well as pharmacological inhibition of Gal-3 significantly reduced capacity of TLR-4 primed DCs to produce immunosuppressive KYN (Figure 5B). TLR-4 priming results in increased activation of Phosphoinositide 3-kinase (PI3K)/Akt signaling pathway in DCs leading to the enhanced production of immunosuppressive cytokines [62]. Activated TLR-4 recruits PI3K that converts phosphatidylinositol 4,5-bisphosphate (PIP2) to phosphatidylinositol 3,4,5-trisphosphate (PIP3). PIP3 enables activation of Akt. Activated Akt, on turn, inactivates Glycogen Synthase Kinase 3 (GSK3) and promotes nuclear accumulation of cAMP Response Element-Binding Protein (CREB) which displaces NF-κB p65 from the co-activator of transcription (CREB binding protein (CBP)). Accordingly, enhanced transcriptional activity of CREB leads to the reduced transcriptional activity of NF-κB p65 and results in increased production of immunosuppressive cytokines and decreased production of pro-inflammatory cytokines in TLR-4-primed DCs [62]. Since Gal-3 stimulates activation of PI3K/Akt signaling pathway in macrophages and promotes their conversion in M2 immunosuppressive phenotype in PI3K/Akt-dependent manner [45], we assume that Gal-3:TLR-4-dependent induction of tolerogenic phenotype in colon infiltrated DCs was a consequence of an increased PI3K/Akt activity.

Gal-3 deficiency abrogated capacity of DCs to prevent transdifferentiation of Tregs in inflammatory Th1 or Th17 cells in IDO-1/KYN dependent manner (Figure 5C). Additionally, Tregs generated by TLR-4 primed Gal-3^−/−^DCs were not able to optimally suppress production of inflammatory cytokines in Th1 and Th17, confirming the importance of Gal-3:TLR-4 interaction for DC-driven regulation of cross-talk between Tregs and effector T cells (Figure 5D). Since LPS preconditioning enhances anti-inflammatory properties of DCs [34], adoptive transfer of TLR-4-primed WTDCs attenuated DSS-induced colitis by increasing serum levels of IL-10 and KYN and by inducing expansion of colon-infiltrating, IL-10-producing Tregs which was accompanied by alleviated Th1 and Th17 cell-driven inflammation (Figure 6). Importantly, this phenomenon was not observed after passive transfer of LPS-primed Gal-3^−/−^DCs, indicating a crucial importance of Gal-3 for TLR-4-dependent activation of IDO-1/KYN pathway in DCs and for DCs-induced expansion of colon-infiltrated Tregs during the alleviation of DSS-induced colitis.

In similar manner as it was observed in animal model, patients’ data indicated important protective role of Gal-3 in alleviation of UC. An increased serum and fecal levels of Gal-3 correlated with concentration of KYN (Figure 8A and Figure 9E) and IL-10 (Figure 8D and Figure 9G) indicating that elevation in Gal-3 reflects overproduction of immunosuppressive cytokines in UC patients. Since up-regulation of KYN and IL-10 in serum and tissue samples of UC patients attenuate detrimental immune response in the gut, alleviate colon inflammation and promote mucosal healing of injured epithelial cells [17], elevated concentration of serum, and fecal Gal-3 may be considered as an additional immunoregulatory mechanism that contributes to the regeneration of the injured gut.

Accordingly, concentration of Gal-3 in serum and stool samples of UC patients negatively correlated with clinical, endoscopic and histological parameters of disease severity (Figure 7C–F). ROC analysis revealed that measurement of serum and fecal levels of Gal-3 (Figure 7G and Figure 9D) could be used as a new diagnostic tool for predicting aggravation of UC, which may complement FC for monitoring attenuation of colon injury in UC patients.

In line with our results, several research groups indicated that serum levels and colon tissue expression of Gal-3 might predict severity and progression of UC [8,13,63]. Frolova and colleagues noticed elevated concentrations of Gal-3 in serum samples of UC patients with active disease [8], while Papa Gobbi and associates revealed dysregulated expression of galectins (Gal-1,-3,-4 and -9) in inflamed colon tissues of IBD patients compared with non-inflamed colon tissue samples of patients suffering from non-inflammatory colon diseases, suggesting that galectin-specific signature in the gut may be used for the diagnosis of Crohn’s disease and UC [13]. Although Papa Gobbi and colleagues managed to discriminate IBD from other intestinal inflammatory conditions by using linear discriminate integrative analysis of galectins, galectin profiling could not distinguish Crohn’s disease and UC-related pathological changes in the gut [13]. By using immunohistochemical analysis, Block and colleagues revealed that Gal-3 is expressed on colon-infiltrated immune cells of UC patients, but its expression on gut epithelial cells did not correlate with disease severity and could not be used for monitoring disease progression [63]. It has to be noted that expression of Gal-3 in serum and colon tissue samples of UC patients is dependent on individual characteristics of UC patients [64], which might be the reason why Cibor and coworkers did not observe a significant difference in serum levels of Gal-3 between UC patients with active and inactive disease [64]. Therefore, heterogeneity of demographic and clinical characteristics of UC patients might affect potential utility of using Gal-3 as a biomarker for monitoring UC progression and have to be considered when serum concentration and expression of Gal-3 in colon tissue are analyzed.

**Figure 10:**
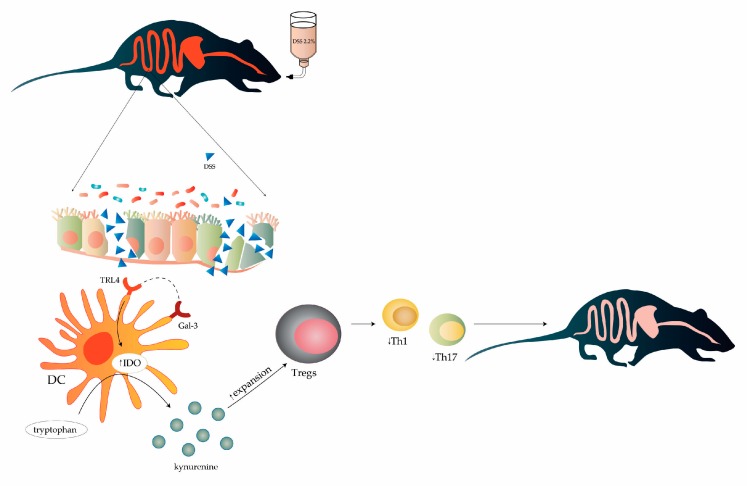
Scheme of Gal-3-dependent modulation of chronic DSS-induced colitis. The main mechanism by which Gal-3 regulates immunosuppressive capacity of regulatory DCs in the gut is relied on the TLR-4-dependent activation of IDO-1/KYN pathway and consequent expansion of colon-infiltrated Tregs, which suppress Th1 and Th17 cell-driven colon inflammation.

In summing up, the main mechanism by which Gal-3 regulates immunosuppressive capacity of regulatory DCs in the gut is relied on the TLR-4-dependent activation of IDO-1/KYN pathway and consequent expansion of colon-infiltrated Tregs which suppress Th1 and Th17 cell-driven colon inflammation (Figure 10). Since serum and fecal levels of Gal-3 inversely reflect disease severity, we proposed that Gal-3 level may serve as a valuable biomarker for monitoring disease progression in UC patients.

## Figures and Tables

**Table 1:** Demographic and clinical characteristics of UC patients.

Characteristic	Mild Chronic Colitis	Moderate Chronic Colitis	Severe Chronic Colitis
Number of patients	39	18	8
Age (range)	26–74	29–82	30–73
Disease location Proctitis/left sided-distal UC/pancolitis	10/23/6	1/10/7	0/4/4
Hb, median (IQR)	134 (8)	109 (18.25)	110.5 (13)
Fe, median (IQR)	14.5 (6.10)	8.15 (6.93)	6.5 (4.4)
Ferritin, median (IQR)	239 (109)	53.5 (102)	20.5 (65.75)
Platelets, median (IQR)	392 (110)	457.5 (124.25)	491.5 (138.25)
UIBC, median (IQR)	44 (13)	32.5 (16.25)	32 (21.5)
TIBC, median (IQR)	52 (12)	56.5 (12.5)	53 (23)
CEA, median (IQR)	1.8 (1.95)	3.75 (2.32)	4.1 (9.59)
CA 19-9, median (IQR)	2.9 (2.4)	10.58 (12.62)	17.82 (21.1)
Disease duration (months) (median; range)	4.4 (1.5–16.5)	6,2 (2.7–10.9)	16.5 (11.4–17.9)
Current therapy AS/CS/IS/IS+BT/BT	38/0/1/0/0	4/0/10/2/2	0/0/4/2/2
Former therapies AS/CS/IS/IS+BT/BT	34/5/0/0/0	2/14/0/1/1	0/6/0/0/2
Per oral steroids at diagnosis Yes/No	5/34	14/4	6/2
Other diseases (Glaucoma/Gonarthrosis)	1/0/	1/1	1/0
Other medications (PG Eye drops/HA)	1/0	1/1	1/0
Smoking status Yes/No	3/36	2/16	0/8

Abbreviations: UC- ulcerative colitis; N- Number; IQR- interquartile range; Hb- hemoglobin; Fe- iron; UIBC-transferrin and iron binding capacity; UIBC-unsaturated iron binding capacity; AS- Aminosalicylates (5-aminosalicylic acid, or 5-ASA); CS- Corticosteroids; IS- Immunosuppressives; BT- Biological therapy (anti-TNF-α monoclonal antibody); PG Eye drops-Prostaglandin Eye drops; HA- Hyaluronic Acid (intra-articular injection).

## References

[1] Strober W., Fuss I., Mannon P. (2007). The fundamental basis of inflammatory bowel disease. J. Clin. Investig..

[2] Naganuma M., Sakuraba A., Hibi T. (2013). Ulcerative colitis: Prevention of relapse. Expert. Rev. Gastroenterol. Hepatol..

[3] Vilela E.G., Torres H.O., Martins F.P., Ferrari M.L., Andrade M.M., Cunha A.S. (2012). Evaluation of inflammatory activity in Crohn’s disease and ulcerative colitis. World J. Gastroenterol..

[4] Chang S., Malter L., Hudesman D. (2015). Disease monitoring in inflammatory bowel disease. World J. Gastroenterol..

[5] Kopylov U., Rosenfeld G., Bressler B., Seidman E. (2014). Clinical utility of fecal biomarkers for the diagnosis and management of inflammatory bowel disease. Inflamm. Bowel. Dis..

[6] Langhorst J., Elsenbruch S., Koelzer J., Rueffer A., Michalsen A., Dobos G.J. (2008). Noninvasive markers in the assessment of intestinal inflammation in inflammatory bowel diseases: Performance of fecal lactoferrin, calprotectin, and PMN-elastase, CRP, and clinical indices. Am. J. Gastroenterol..

[7] Ministro P., Martins D. (2017). Fecal biomarkers in inflammatory bowel disease: How, when and why?. Expert Rev. Gastroenterol. Hepatol..

[8] Frolová L., Smetana K., Borovská D., Kitanovicová A., Klimesová K., Janatková I., Malícková K., Lukás M., Drastich P., Benes Z. (2009). Detection of galectin-3 in patients with inflammatory bowel diseases: New serum marker of active forms of IBD?. Inflamm. Res..

[9] Simovic Markovic B., Nikolic A., Gazdic M., Bojic S., Vucicevic L., Kosic M., Mitrovic S., Milosavljevic M., Besra G., Trajkovic V. (2016). Galectin-3 Plays an Important Pro-inflammatory Role in the Induction Phase of Acute Colitis by Promoting Activation of NLRP3 Inflammasome and Production of IL-1β in Macrophages. J. Crohn’s Colitis.

[10] Tsai H.F., Wu C.S., Chen Y.L., Liao H.J., Chyuan I.T., Hsu P.N. (2016). Galectin-3 suppresses mucosal inflammation and reduces disease severity in experimental colitis. J. Mol. Med..

[11] Lippert E., Stieber-Gunckel M., Dunger N., Falk W., Obermeier F., Kunst C. (2015). Galectin-3 Modulates Experimental Colitis. Digestion.

[12] Müller S., Schaffer T., Flogerzi B., Fleetwood A., Weimann R., Schoepfer A.M., Seibold F. (2006). Galectin-3 modulates T cell activity and is reduced in the inflamed intestinal epithelium in IBD. Inflamm. Bowel. Dis..

[13] Papa Gobbi R., De Francesco N., Bondar C., Muglia C., Chirdo F., Rumbo M., Rocca A., Toscano M.A., Sambuelli A., Rabinovich G.A. (2016). A galectin-specific signature in the gut delineates Crohn’s disease and ulcerative colitis from other human inflammatory intestinal disorders. Biofactors.

[14] Prakash N., Stumbles P., Mansfield C. (2013). Initial validation of cytokine measurement by ELISA in canine feces. Open J. Vet. Med..

[15] Ling W., Zhang J., Yuan Z., Ren G., Zhang L., Chen X., Rabson A.B., Roberts A.I., Wang Y., Shi Y. (2014). Mesenchymal stem cells use IDO to regulate immunity in tumor microenvironment. Cancer Res..

[16] Kostas A., Siakavellas S.I., Kosmidis C., Takou A., Nikou J., Maropoulos G., Vlachogiannakos J., Papatheodoridis G.V., Papaconstantinou I., Bamias G. (2017). Fecal calprotectin measurement is a marker of short-term clinical outcome and presence of mucosal healing in patients with inflammatory bowel disease. World J. Gastroenterol..

[17] Acovic A., Simovic Markovic B., Gazdic M., Arsenijevic A., Jovicic N., Gajovic N., Jovanovic M., Zdravkovic N., Kanjevac T., Harrell C.R. (2018). Indoleamine 2,3-dioxygenase-dependent expansion of T-regulatory cells maintains mucosal healing in ulcerative colitis. Therap. Adv. Gastroenterol..

[18] Volarevic V., Milovanovic M., Ljujic B., Pejnovic N., Arsenijevic N., Nilsson U., Leffler H., Lukic M.L. (2012). Galectin-3 Deficiency Prevents Concanavalin A-Induced Hepatitis in Mice. Hepatology.

[19] Rahman A., Fahlgren A., Sundstedt C., Hammarström S., Danielsson A., Hammarström M.L. (2011). Chronic colitis induces expression of β-defensins in murine intestinal epithelial cells. Clin. Exp. Immunol..

[20] Murthy S.N., Cooper H.S., Shim H., Shah R.S., Ibrahim S.A., Sedergran D.J. (1993). Treatment of dextran sulfate sodium-induced murine colitis by intracolonic cyclosporine. Dig. Dis. Sci..

[21] Whittem C.G., Williams A.D., Williams C.S. (2010). Murine Colitis Modeling using Dextran Sulfate Sodium. J. Vis. Exp..

[22] Obermeier F., Kojouharoff G., Hans W., Schölmerich J., Gross V., Falk W. (1999). Interferon-gamma (IFN-gamma)- and tumour necrosis factor (TNF)-induced nitric oxide as toxic effector molecule in chronic dextran sulphate sodium (DSS)-induced colitis in mice. Clin. Exp. Immunol..

[23] Yanagawa Y., Onoé K. (2007). Enhanced IL-10 production by TLR4- and TLR2-primed dendritic cells upon TLR restimulation. J. Immunol..

[24] Mizuno N., Sasaki Y., Segawa R., Asakawa S., Hiratsuka M., Hirasawa N. (2018). LPS priming in early life decreases antigen uptake of dendritic cells via NO production. Immunobiology.

[25] Demotte N., Bigirimana R., Wieërs G., Stroobant V., Squifflet J.L., Carrasco J., Thielemans K., Baurain J.F., Van Der Smissen P., Courtoy P.J. (2014). A short treatment with galactomannan GM-CT-01 corrects the functions of freshly isolated human tumor-infiltrating lymphocytes. Clin. Cancer Res..

[26] Abe K., Nguyen K.P., Fine S.D., Mo J.H., Shen C., Shenouda S., Corr M., Jung S., Lee J., Eckmann L. (2007). Conventional dendritic cells regulate the outcome of colonic inflammation independently of T cells. Proc. Natl. Acad. Sci. USA.

[27] Kourepini E., Aggelakopoulou M., Alissafi T., Paschalidis N., Simoes D.C., Panoutsakopoulou V. (2014). Osteopontin expression by CD103- dendritic cells drives intestinal inflammation. Proc. Natl. Acad. Sci. USA.

[28] Tahara M., Kondo Y., Yokosawa M., Tsuboi H., Takahashi S., Shibayama S., Matsumoto I., Sumida T. (2015). T-bet regulates differentiation of forkhead box protein 3+ regulatory T cells in programmed cell death-1-deficient mice. Clin. Exp. Immunol..

[29] Sharma M.D., Baban B., Chandler P., Hou D.Y., Singh N., Yagita H., Azuma M., Blazar B.R., Mellor A.L., Munn D.H. (2007). Plasmacytoid dendritic cells from mouse tumor-draining lymph nodes directly activate mature Tregs via indoleamine 2,3-dioxygenase. Proc. J. Clin. Investig..

[30] Saksida T., Nikolic I., Vujicic M., Nilsson U.J., Leffler H., Lukic M.L., Stojanovic I., Stosic-Grujicic S. (2013). Galectin-3 deficiency protects pancreatic islet cells from cytokine-triggered apoptosis In Vitro. J. Cell. Physiol..

[31] Dieleman L.A., Ridwan B.U., Tennyson G.S., Beagley K.W., Bucy R.P., Elson C.O. (1994). Dextran sulfate sodium-induced colitis occurs in severe combined immunodeficient mice. Gastroenterology.

[32] Melgar S., Karlsson A., Michaëlsson E. (2005). Acute colitis induced by dextran sulfate sodium progresses to chronicity in C57BL/6 but not in BALB/c mice: Correlation between symptoms and inflammation. Am. J. Physiol. Gastrointest. Liver Physiol..

[33] Kim T.W., Seo J.N., Suh Y.H., Park H.J., Kim J.H., Kim J.Y., Oh K.I. (2006). Involvement of lymphocytes in dextran sulfate sodium-induced experimental colitis. World J. Gastroenterol..

[34] Salazar F., Awuah D., Negm O.H., Shakib F., Ghaemmaghami A.M. (2017). The role of indoleamine 2,3-dioxygenase-aryl hydrocarbon receptor pathway in the TLR4-induced tolerogenic phenotype in human DCs. Sci. Rep..

[35] Matteoli G., Mazzini E., Iliev I.D., Mileti E., Fallarino F., Puccetti P., Chieppa M., Rescigno M. (2010). Gut CD103+ dendritic cells express indoleamine 2,3-dioxygenase which influences T regulatory/T effector cell balance and oral tolerance induction. Gut.

[36] Abraham B.P., Kane S. (2012). Faecal markers: Calprotectin and lactoferrin. Gastroenterol. Clin. N. Am..

[37] Fu Y., Wang L., Xie C., Zou K., Tu L., Yan W., Hou X. (2017). Comparison of non-invasive biomarkers faecal BAFF, calprotectin and FOBT in discriminating IBS from IBD and evaluation of intestinal inflammation. Sci. Rep..

[38] Mak W.Y., Buisson A., Andersen M.J., Lei D., Pekow J., Cohen R.D., Kahn S.A., Pereira B., Rubin D.T. (2018). Fecal Calprotectin in Assessing Endoscopic and Histological Remission in Patients with Ulcerative Colitis. Dig. Dis. Sci..

[39] Bauer C., Duewell P., Mayer C., Lehr H.A., Fitzgerald K.A., Dauer M., Tschopp J., Endres S., Latz E., Schnurr M. (2010). Colitis induced in mice with dextran sulfate sodium (DSS) is mediated by the NLRP3 inflammasome. Gut.

[40] Neuman M.G. (2004). Signaling for inflammation and repair in inflammatory bowel disease. Rom. J. Gastroenterol..

[41] Trivedi P.J., Adams D.H. (2018). Chemokines and Chemokine Receptors as Therapeutic Targets in Inflammatory Bowel Disease; Pitfalls and Promise. J. Crohns Colitis.

[42] McAlindon M.E., Hawkey C.J., Mahida Y.R. (1998). Expression of interleukin 1 beta and interleukin 1 beta converting enzyme by intestinal macrophages in health and inflammatory bowel disease. Gut.

[43] Isidro R.A., Appleyard C.B. (2016). Colonic macrophage polarization in homeostasis, inflammation, and cancer. Am. J. Physiol. Gastrointest. Liver Physiol..

[44] Kojouharoff G., Hans W., Obermeier F., Männel D.N., Andus T., Schölmerich J., Gross V., Falk W. (1997). Neutralization of tumour necrosis factor (TNF) but not of IL-1 reduces inflammation in chronic dextran sulphate sodium-induced colitis in mice. Clin. Exp. Immunol..

[45] MacKinnon A.C., Farnworth S.L., Hodkinson P.S., Henderson N.C., Atkinson K.M., Leffler H., Nilsson U.J., Haslett C., Forbes S.J., Sethi T. (2008). Regulation of alternative macrophage activation by galectin-3. J. Immunol..

[46] Matricon J., Barnich N., Ardid D. (2010). Immunopathogenesis of inflammatory bowel disease. Self Nonself.

[47] Yamada A., Arakaki R., Saito M., Tsunematsu T., Kudo Y., Ishimaru N. (2016). Role of regulatory T cell in the pathogenesis of inflammatory bowel disease. World J. Gastroenterol..

[48] Wu W., Chen F., Liu Z., Cong Y. (2016). Microbiota-specific Th17 Cells: Yin and Yang in Regulation of Inflammatory Bowel Disease. Inflamm. Bowel. Dis..

[49] Nikolaus S., Schulte B., Al-Massad N., Thieme F., Schulte D.M., Bethge J., Rehman A., Tran F., Aden K., Häsler R. (2017). Increased Tryptophan Metabolism Is Associated with Activity of Inflammatory Bowel Diseases. Gastroenterology.

[50] Etienne-Mesmin L., Chassaing B., Gewirtz A.T. (2017). Tryptophan: A gut microbiota-derived metabolites regulating inflammation. World J. Gastrointest. Pharmacol. Ther..

[51] Islam J., Sato S., Watanabe K., Watanabe T., Hirahara K.A., Aoyama Y., Tomita S., Aso H., Komai M., Shirakawa H. (2017). Dietary tryptophan alleviates dextran sodium sulfate-induced colitis through aryl hydrocarbon receptor in mice. J. Nutr. Biochem..

[52] Shizuma T., Mori H., Fukuyama N. (2013). Protective effect of tryptophan against dextran sulfate sodium- induced experimental colitis. Turk. J. Gastroenterol..

[53] Boasso A., Vaccari M., Hryniewicz A., Fuchs D., Nacsa J., Cecchinato V., Andersson J., Franchini G., Shearer G.M., Chougnet C. (2007). Regulatory T-cell markers, indoleamine 2,3-dioxygenase, and virus levels in spleen and gut during progressive simian immunodeficiency virus infection. J. Virol..

[54] Acovic A., Gazdic M., Jovicic N., Harrell C.R., Fellabaum C., Arsenijevic N., Volarevic V. (2018). Role of indoleamine 2,3-dioxygenase in pathology of the gastrointestinal tract. Therap. Adv. Gastroenterol..

[55] Zhang X.J., Yuan Z.W., Qu C., Yu X.T., Huang T., Chen P.V., Su Z.R., Dou Y.X., Wu J.Z., Zeng H.F. (2018). Palmatine ameliorated murine colitis by suppressing tryptophan metabolism and regulating gut microbiota. Pharmacol. Res..

[56] Ferdinande L., Demetter P., Perez-Novo C., Waeytens A., Taildeman J., Rottiers I., Rottiers P., De Vos M., Cuvelier C.A. (2008). Inflamed intestinal mucosa features a specific epithelial expression pattern of indoleamine 2,3-dioxygenase. Int. J. Immunopathol. Pharmacol..

[57] Furuzawa-Carballeda J., Fonseca-Camarillo G., Lima G., Yamamoto-Furusho J.K. (2013). Indoleamine 2,3-dioxygenase: Expressing cells in inflammatory bowel disease-a cross-sectional study. Clin. Dev. Immunol..

[58] Fougeray S., Mami I., Bertho G., Beaune P., Thervet E., Pallet N. (2012). Tryptophan depletion and the kinase GCN2 mediate IFN-γ-induced autophagy. J. Immunol..

[59] Metz R., Rust S., Duhadaway J.B., Mautino M.R., Munn D.H., Vahanian N.N., Link C.J., Prendergast G.C. (2012). IDO inhibits a tryptophan sufficiency signal that stimulates mTOR: A novel IDO effector pathway targeted by D-1-methyl-tryptophan. Oncoimmunology.

[60] Munn D.H., Mellor A.L. (2016). IDO in the Tumor Microenvironment: Inflammation, Counter-Regulation, and Tolerance. Trends Immunol..

[61] Deierborg T., Burguillos M.A. (2015). A new “sweet” ligand for Toll-like receptor 4. Oncotarget.

[62] Brown J., Wang H., Hajishengallis G.N., Martin M. (2011). TLR-signaling networks: An integration of adaptor molecules, kinases, and cross-talk. J. Dent. Res..

[63] Block M., Mölne J., Leffler H., Börjesson L., Breimer M.E. (2016). Immunohistochemical Studies on Galectin Expression in Colectomised Patients with Ulcerative Colitis. Biomed. Res. Int..

[64] Cibor D., Szczeklik K., Brzozowski B., Mach T., Owczarek D. (2019). Serum galectin 3, galectin 9 and galectin 3-binding proteins in patients with active and inactive inflammatory bowel disease. J. Physiol. Pharmacol..

